# Relationship between inspiratory muscle strength and balance in women: A cross-sectional study

**DOI:** 10.1371/journal.pone.0280465

**Published:** 2023-02-15

**Authors:** Ingrid Guerra Azevedo, Saionara M. A. da Câmara, Alvaro C. C. Maciel, Edgar R. Vieira, Camila F. L. Espinoza, Sebastián M. M. Pichún, Elizabel de S. R. Viana, Silvana L. de O. Sousa

**Affiliations:** 1 Departamento de Procesos Terapéuticos, Universidad Católica de Temuco UCT, La Araucanía, Temuco, Chile; 2 Departamento de Fisioterapia, Federal University of Rio Grande do Norte UFRN, Natal, Rio Grande do Norte (RN), Brazil; 3 Department of Physical Therapy, Florida International University, Miami, Florida, United States of America; 4 Facultad de Medicina, Universidad de Murcia, Murcia, España; Universitat de Valencia, SPAIN

## Abstract

**Background:**

There is scarce evidence on changes at the functional level associated with the respiratory area in women. This study aims to analyse the relationship between inspiratory muscle strength and balance in women.

**Material and methods:**

In this cross-sectional observational study, the sample consisted of groups according to the results obtained in the balance test. Inspiratory muscle weakness was defined as maximum inspiratory pressure (MIP) ≤ 80% of the predictive value. MIP was carried out using through a mouthpiece, with an electronic manometer. Logistic regression model was used to examine if MIP predicts balance.

**Results:**

159 women participated in the study. Approximately 20% of them achieved balance ≤ 2 seconds and 18% presented MIP≤80%. MIP was associated with the time achieved in the one-leg support test. Subjects with MIP ≤ 80% of the predictive value show 3 times more risk of having a lower performance in the balance test (OR = 3.26).

**Conclusions:**

Inspiratory muscle weakness is associated with deficient balance in this sample. It shows the need for multidimensional assessment and rehabilitation strategies for patients identified as having MIP weakness and/or balance disorders.

## Introduction

By the year of 2030, 1 in 6 people in the world will be aged 60 years or over. By 2050, the world’s population of people aged 60 years and older is expected to increase to 2.1 billion [1]. Additionally, women outnumber men at older ages [2]. Currently, in 2022, women comprised 55.7 percent of persons aged 65 or older [2].

As people age, they experience progressive physical alterations and functional deterioration [3]. Some of these alterations occur in the musculoskeletal system, leading to reduced muscle strength or power, such as dynapenia and sarcopenia, giving rise to functional changes such as slower reflexes, reduced mobility or deficient postural balance [3]. Postural balance is defined as the ability of an individual to maintain equilibrium during both static and dynamic tasks [4]. Balance, particularly, can be divided into two types, static, which is responsible for the ability to maintain an upright posture and to keep the line of gravity within the limits of the base of support, and dynamic, which is related to the ability to maintain stability activities involving changing the base of support [4].

Falls can occur in both static and dynamic situations and disorders in both types of balance are known risk factors for falls among older people [4]. Furthermore, the presence of dynapenia and sarcopenia lead to reduced muscle strength or power, increasing the risk of functional deterioration, falls and mortality [5]. Statistical data confirm that accidental falls are the third most prevalent cause of death, and the primary cause of non-fatal accidents in this population [6]. In Brazil, between the period from 2000 to 2019, a total of 135,209 deaths resulting from falls in older adults has been identified [7]. Mortality from falls in general, during the aforementioned period, had an upward trend in older adults of both sexes [7]. In the respiratory system, aging is associated with biological changes to both, lung structures and thorax (including the respiratory muscles), which increase the work of breathing and impair the full performance of its function [8]. In particular, the inspiratory muscles go through a gradual decline in inspiratory muscle strength and mass with age [8, 9]. In comparison with younger adults, the elderly present a reduction of up to 25% in diaphragm strength [9]. Additionally, with age, the lungs tissues undergo changes with that lead to an increase in alveolar size, which lowers the alveolar surface tension and so reduces the elastic recoil of the lungs [8]. Then, it causes a reduction in maximum achievable flow in the airways during the breathing cycle. Hence, in older people, the lower muscles’ performance, the increased alveolar size and the stiffer chest wall, lead altogether to an increased residual volume and also counter the possible increase in total lung capacity from the reduced elastic recoil [8, 9].

In women, menopause causes hormonal changes and systemic alterations [10, 11]. These hormonal alterations cause a decrease in muscle strength, decreased relaxation of bronchial smooth muscle and increased compression of the thoracic spine due to osteoporosis, directly interfering with the functioning of the pulmonary system [10]. Still related to women’s health particularities, there is also evidence that multiparity is related to limited inspiratory strength, where the higher the number of pregnancies, the lower the values of maximal inspiratory pressure [12]. All these aspects show how inspiratory muscles strength may be influenced by different female health variables and could be associated with others.

This weakness of the respiratory muscles affects not only the respiratory function, but other ones as well [13–15], since the respiratory muscles have a double role, playing a part in postural stabilisation [16, 17]. The diaphragm, together with the abdominal muscles and the pelvic floor, work in coordination to increase the intra-abdominal pressure and provide stability to the lower trunk before the execution of voluntary tasks [16, 17]. Thus, diminished inspiratory muscle strength may develop into a deterioration of physical capacity on one hand [14, 15] and affect postural balance and risk of falling on the other [17]. Moreover, balance disorders and respiratory muscles weakness can share similar causes, which makes both be associated with each other. Ageing related disorders leading to muscle deterioration can affect both peripheral and respiratory muscles [13–15], and early identification of people at risk of such conditions is important for preventive purposes.

Although there is certain evidence in the literature that increasing the efficiency of respiratory muscles improves postural balance in elderly people [17–20], few investigations have been carried out considering the relationship between postural balance and objective measurements (such as maximal respiratory pressures) of muscle strength in exclusively female populations [17, 21] associated with functions altered by the changes occurring during the aging process, and in addition to the subject’s reproductive history [11, 12, 15]. So, it is of extreme importance to develop research in this specific field in order to direct the assessments and consequently, the treatments for this population, considering objective evaluation techniques.

We hypothesize that the weaker the maximal inspiratory pressures (<80% of the normal predictive value), the worse the postural balance in women aged between 41 and 80 years old, from Northeast Brazil. Thus, the object of this study was to investigate the relationship between inspiratory muscle strength and postural balance in middle-aged and elderly women resident in the community.

## Materials and methods

A cross-sectional observational study was designed and carried out in the period April to August 2016, in a city in Northeast Brazil (Santa Cruz, Rio Grande do Norte), population 36,674.

### Population and sample size

Participants were excluded if they presented: cognitive deterioration, defined as four or more mistakes on the Leganés cognitive test orientation scale, since this was considered an indication that they would not be able to complete the study protocols [22]; inability to carry out maximum respiration manoeuvres; labyrinthitis assessed through self-reporting. Additionally, we excluded women with neurological impairments, degenerative diseases, or any chronic condition that could compromise the respiratory muscle strength evaluation and musculoskeletal assessment.

A convenience sample size was used, and the subjects were invited to participate through a campaign in the city’s health centres, senior citizen centres and hospitals. This campaign was part of a wider investigation, the initial object of which was to assess the relationship between female reproductive variables and respiratory muscle strength [12, 15]. The sample size calculated initially was 200 subjects. The sample size was estimated from the larger prospective study based on the rule of thumb that at least 15 subjects per predictor are needed for a reliable equation in multiple regression models [23]. Thus, 200 subjects were recruited, assuming a maximum of 10 predictors and 20 subjects per predictor. Fifty women were recruited for each of the age bands of interest in the study to guarantee relatively even representation of participants in each age bracket (41–50; 51–60; 61–70; 71–80 years) [12].

**Fig 1:**
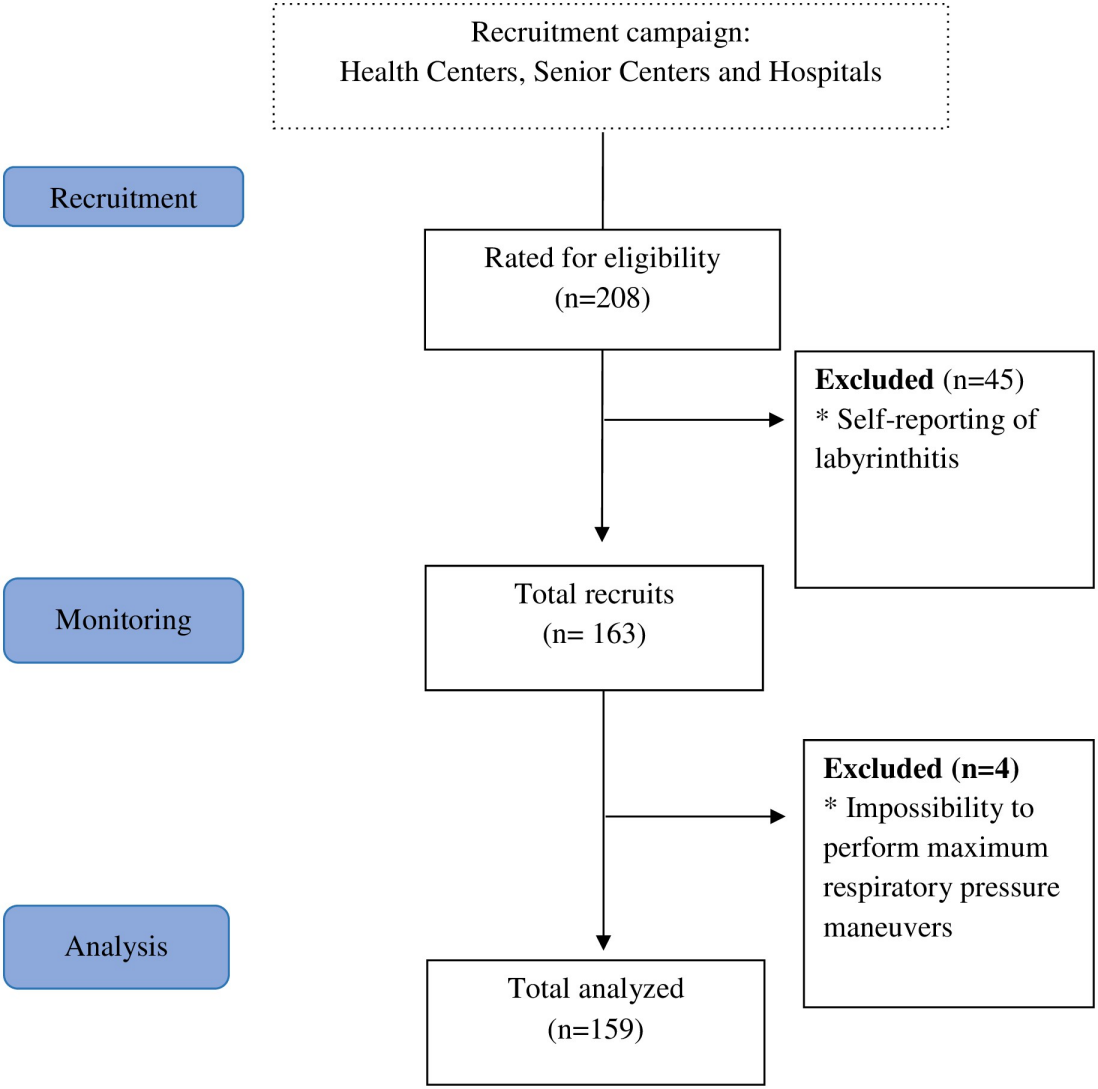
Flowchart explaining the participants progression, from sample recruitment to analysis.

The women recruited into the study were aged between 41 and 80 years, with a mean physical activity level of 37 minutes in the week previous to the evaluation; a total of 208 women interested in participating in the study attended the research centre. Of these, 49 were excluded (mean age 61.6 years) for self-reported labyrinthitis, and 159 finally took part in the study. The process for selecting the participants is shown in Fig 1.

### Ethical aspects

The study was approved by the Ethics and Research Committee of the Federal University of Rio Grande do Norte, in the year of 2015 (No.1875802). All the participants were informed of the objectives and procedures of the study during the first contact; all of them signed an informed consent document before their data was recorded.

### Procedures

All the assessment procedures were carried out by previously trained investigators in a laboratory of the Centre for Health Sciences Research—Federal University of Rio Grande do Norte (Santa Cruz, Rio Grande do Norte, Brazil). The laboratory is specifically equipped to carry out physical and respiratory function tests.

The measurements were made in the same day and the order of the tests was:

1. Participant identification
2. Leganés cognitive test orientation scale
3. Associated Illnesses
4. Lifestyle (smoking, physical activity)
5. Physical Evaluation (BMI, Knee extension force, postural balance)
6. Respiratory Muscle Strength (Maximum Inspiratory Pressure)

### Dependent variable

#### Postural balance

To assess one-leg balancing, the participant was asked to balance on the dominant leg, identified by self-report. The time that this position was maintained was recorded, up to a maximum of 30 seconds. The test was carried out first with eyes open and then with eyes closed [13, 24]. The variable used for analysis was the mean of three tests with eyes closed. The result was divided into two groups based on the ability to maintain balance on one leg with eyes closed (≤ 2 seconds; > 2 seconds), which was considered a categorical variable. These categories were used to separate the participants by ability to perform the balancing test. We have not located in the literature studies suggesting cut-off points for one-leg support time with closed eyes related to balance disorders. The studies identified considered the values of this test, but with open eyes.

Different cut-off points for balance impairment, such as values <1.02 sec [25], <3.0seg [25], <8.0seg [26], have been described for the prediction of falls. There are data on the normative values of both tests, with open and closed eyes, according to age and sex groups [27]. Thus, based on the normative values and the cut-off points of this test for the prediction of falls with open eyes, we have chosen the value of 2 seconds to classify our patients with better or worse balance.

### Independent variable

#### Maximum inspiratory pressure

The maximum inspiratory pressure was measured using an MVD 300^®^ electronic manometer (-300 to +300 cmH20) (Globalmed^®^, Porto Alegre, Rio Grande do Sul, Brazil). The volunteers adopted the sitting position [28]. They were asked to exhale to the maximum possible (close to residual volume), and then carry out maximum inspiration (close to total lung capacity) to allow the maximum inspiratory pressure (MIP) to be assessed. The highest value obtained in the three best attempts with a difference of no more than 10% was selected as the MIP value [28]. A nasal clip was used during the maneuver performance [28]. The MIP value was categorised into two groups: ≤ 80% and >80% of the predictive value for women of the Brazilian population according to the Neder predictive equation (MIP: y = -0.49 (age) + 110.4, SEE = 9.1. 22) [29].

### Co-variables

#### Age

Age was considered as a co-variable in these analyses and was used as a continuous quantitative measurement.

#### Body mass index (BMI)

The BMI was calculated from the measured weight (kg) and height (m), using the formula: BMI = weight/height^2^ (kg/m^2^). This variable was used in the analysis as a continuous quantitative measurement [30].

#### Physical activity

Using a questionnaire specially constructed for this study, the participants were asked how many days and how often per day they had walked continuously for more than 10 minutes during the previous week. This measurement included walking as a means of getting from one place to another or as exercise [12]. This variable was used in the analysis as a continuous quantitative measurement.

#### Smoking

The participants were asked about their consumption of tobacco. The sample was divided into "never a smoker" and "smoker now or in the past".

#### Knee extension force

The knee extension force was assessed using a model MicroFET2 portable dynamometer (Hoggan^®^ Health Industries, UT, USA), which records muscle force in kilograms/force (Kgf). The participants sat on a bench with legs dangling with knees flexed at 90 degrees and hands on thighs. The dynamometer was positioned by the interviewer in the anterior region of the distal leg, just above the malleolus line [31]. The participant was requested to apply her maximum strength against the dynamometer, per five seconds, as she would extend the knees. An inelastic ribbon attached to the wall helped the interviewer to keep the dynamometer in the same position during the assessment, to allow the isometric muscle contraction. The value taken was the mean of three extension tests with the dominant leg with one-minute interval between measurements. This variable was used in the analysis as a continuous quantitative measurement.

### Data analysis

The values were entered into a database and statistical analyses were carried out using the *Statistical Package for the Social Sciences* (SPSS) version 22.0 (IBM, Chicago, IL, USA). The Kolmogorov-Smirnov test was used to assess the normality of the distribution of variables. The continuous variables were expressed as mean ± standard deviation. The categorical variables were expressed as percentages and absolute numbers.

The characteristics of the sample were presented in accordance with the independent variable. The Chi-squared test was used to compare categorical variables. The continuous variables were analysed using the Student’s t-test. We created a binary logistic regression model to evaluate the association between postural balance and the percentage of MIP predictive value, adjusted for the co-variables (age, BMI, smoking, physical activity and knee extension force). The backward method was used and variables with statistically significant associations (p <0.05) with postural balance and MIP remained in the multivariate model. The threshold of significance was set at 5%, with CI 95%, for all analyses.

## Results

A total of 159 women aged 41–80 years participated in the study, being 39 women from 41–50; 42 women from 51–60; 40 women from 61–70 and 38 from 71–80 years old The 49 excluded subjects were distributed among the following age range: 13 women from 41–50; 10 from 51–60; 14 from 61–70 and 12 from 71–80 years old.

Table 1 shows the socio-demographic and clinical characteristics (co-variables) of the participants, grouped by presence (≤80% predictive MIP) or absence (>80% predictive MIP) of inspiratory muscle weakness. The mean age of the subjects presented a statistically significant difference, showing that women with MIP ≤ 80% of the predictive value presented a higher mean age than the group of women with MIP >80% of the predictive value. Likewise, a statistically significant difference was observed for the knee extension force (p = 0.018) and for postural balance (p = 0.003); the group of women with MIP >80% presented a higher knee extension force and higher absolute postural balance values. The percentage of women with MIP >80% of the predictive value was higher in the group that had never smoked (63.8%) than in the subjects who stated that they were recurrent or ex-smokers (36.2%), presenting a statistically significant difference (p = 0.026). No statistically significant difference between the groups was recorded for the variables BMI and physical activity.

**Table 1:** Characteristics of the participants by MIP predictive value.

Variable	Total sample (n = 159)	>80%MIP (n = 130)	≤80%MIP (n = 29)	*p* value
Age (years)	59.71 (11.27)	54.27 (10.9)	58.69 (11.1)	0.015^a^
BMI (kg/m^2^)	28.83 (4.56)	28.28 (4.1)	28.96 (4.6)	0.469^a^
Physical activity (min)	37.33 (43.53)	35.33 (40.5)	38.33 (44.2)	0.713^a^
KEF (Kgf)	20.53 (7.78)	21.22 (7.9)	17.44 (6.4)	0.018^a^
Postural Balance (sec)	5.87 (5.68)	6.56 (0.52)	3.04 (0.49)	0.003 ^a^
Smoker				
*Never*	95 (59.7%)	83 (63.8%)	12 (41.4%)	0.026^b^
*Recurrent or ex-smoker*	64 (40.3%)	47 (36.2%)	17 (58.6%)	

Data presented as mean (SD) or n (%)

BMI: body mass index; kg/m^2^: kilogram per square metre; min: minutes; sec: seconds; KEF: Knee extension force; MIP: Maximum inspiratory pressure

Kgf: kilograms force.

^a^: Student’s t-test /

^b^Chi-squared Test

In Table 2 we can identify the characteristics of the participants associated with their balance values (≤ 2 or > 2 seconds). Women in the group with balance > 2 seconds were younger, with greater knee extension force and MIP > 80% of the predictive value; the p-values were statistically significant. When considering absolute values, the group with balance > 2 seconds presented higher mean MIP values (112.9 mmHg) than the MIP values achieved by the group with ≤ 2 seconds (83.6 mmHg), p <0.001. Likewise, a higher percentage of women with better balance reported that they had never smoked (64.6%), as compared to subjects who were recurrent or ex-smokers (35.4%). Furthermore, the percentage of women with better postural balance was higher in the group of subjects with MIP >80% of the predictive value (87.4%); a statistically significant difference was observed with the women with MIP ≤ 80% of predictive values (p <0.001).

**Table 2:** Characteristics of the participants by postural balance scores.

Variable	> 2 seconds (n = 127)	≤ 2 seconds (n = 32)	*p* value
Age (years)	57.63 (10.6)	67.93 (10.0)	<0.001^a^
BMI (kg/m^2^)	28.94 (4.6)	28.40 (4.3)	0.551^a^
Physical activity (min)	37.24 (42.5)	39.29 (47.8)	0.777 ^a^
KEF (Kgf)	21.50 (7.9)	16.68 (5.6)	0.002^a^
Smoker			
*Never*	82 (64.6%)	13 (40.6%)	0.014^b^
*Recurrent or ex-smoker*	45 (35.4%)	19 (59.4%)	
MIP			<0.001^b^
*≤ 80% MIP*	16 (12.6%)	13 (40.6%)	
*>80% MIP*	111 (87.4%)	19 (59.4%)	
MIP* (mmHg)	112.9 (4.1)	83.6 (6.8)	<0.001^a^

Data presented as mean (SD) or n (%)

BMI: body mass index; kg/m^2^: kilogram per square metre; min: minutes; KEF: Knee extension force; MIP: Maximum inspiratory pressure—categories for predicted values

Kgf: kilograms force.

MIP*: Absolut values for maximum inspiratory pressure; mmHg: millimetres of mercury.

^a^: Student’s t-test /

^b^Chi-squared Test

Table 3 allows us to identify variables associated with postural balance adjusted for the co-variables. Age presents a positive Odds Ratio (OR = 1.07), while each additional unit of knee extension force is associated with a reduction of 7% in the risk of presenting a deterioration in postural balance (≤ 2 seconds) (OR = 0.93). The same behaviour is observed in the MIP, where the less than or equal to 80% of the predictive value increases to three times the risk of having a lower performance in the one-leg balancing test (OR = 3.26; p = 0.007).

**Table 3:** Binary logistic regression for variables associated with postural balance.

Variables	Standard Error	OR	p-value	IC95%
**Age (years)**	0.020	1.07	<0.001	1.03–1.12
**KEF (Kgf)**	0.033	0.93	0.024	0.87–0.99
**MIP**	-	-	-	-
*>80%*	0.437	1	-	-
*≤80%*	-	3.26	0.007	1.38–7.69
**Constant**	1.531	0.008	0.001	-

MIP: Maximum inspiratory pressure; KEF: Knee extension force

Nagelkerke R Square: 0.28

Hosmer and Lemeshow Test: p = 0.41

## Discussion

The results of this study show that inspiratory muscle strength is associated with postural balance in middle-aged and elderly women. Having a maximum inspiratory pressure (MIP) below 80% of the normal predictive value increases the risk of presenting deficient postural balance (≤ 2 seconds in one-leg balancing) by three times (OR = 3.26). This association was maintained even after adjusting for such important co-variables for balance such as age and knee extension force. Based on that, our main finding support the initial hypothesis, since it was shown that the lower the MIP, the worse the postural balance in healthy middle aged and older women.

Our results corroborate the hypothesis proposed initially by Hodges et al [32], and subsequently by other authors [18], that the diaphragm plays a role in modulating postural control and balance [18]; the latter authors showed that inspiratory muscle weakness contributes to balance deficiencies in everyday activities, such as getting up from a chair [19].

In a geriatric population, it was shown that respiratory muscle strength was an independent predictor of the decline in mobility of elderly residents in the community, after adjusting for lower limbs strength and physical activity [33]. This corroborates with our results, since that in our sample, the inspiratory muscle weakness and deficient postural balance were independent of the physical activity level, supporting the idea that global physical exercise does not affect the respiratory muscles. Hence, our findings point to a need for multiprofessional assessment and rehabilitation strategies for women identified as having MIP weakness and/or balance disorders, initiating in middle aged women, in order to mitigate falls in older adults. In Brazil, between the years of 2000 and 2019, there was an upward trend in mortality rates caused by falls [7], evidencing the importance of focusing on promoting health in older adults and preventing the risk of falls.

Although studies agree on a clear relationship of the diaphragm with balance and mobility modulation [32, 33], the mechanisms underlying this relationship remain uncertain. The most widely accepted theory to explain the participation of the diaphragm in postural control is that diaphragm contraction and core muscles work in coordination to increase intra-abdominal pressure (IAP) [25, 34] and provide stability to the lower trunk during static postures or in anticipation of rapid voluntary movements [32].

When the diaphragm is weak, the length-tension ratio is reduced, resulting in reduced diaphragm movement, and thus insufficient IAP [3, 35]. Insufficient IAP may cause poor stimulation of the proprioceptors located in the crural region of the diaphragm, and consequently may disturb the projection of proprioceptive information to the central nervous system. This information is necessary for optimal postural control and balance [36]. We can therefore assume that both weakness and incorrect use of the diaphragm (predominantly thoracic respiratory pattern) may cause deficient balance.

In conjunction with rectus abdominis, the diaphragm is activated in a feedforward manner, theoretically to aid balance during rapid and destabilising movements [37]. Once this muscle presents weakness, there is a failed coordination of the upper-body and lower-body segmental linkage, diminishing the ability to increase IAP, and, consequentially, causing a poor postural balance [38]. All of this support one more time the importance of assessing and training specifically the respiratory muscles in populations going through their ageing process.

The presence of inspiratory muscle weakness in the sample was over 18% of the participants. The group with balance > 2 seconds was younger and exhibited higher mean MIP values (112.9 mmHg) than the MIP values displayed by the group with ≤ 2 seconds (83.6 mmHg). Additionally, the group with ≤ 80%MIP presented a higher mean age than the group with >80%MIP, i.e, the group considered to present inspiratory muscle weakness was older than the one with >80%MIP (54.2 x 58.7 years, respectively, p = 0.015). This data corroborates with the evidence that postural balance deteriorates with advancing age, and the great majority of elderly people are at risk of falling during everyday activities [14]. Moreover, it also coincides with the evidence that MIP values become lower with advancing age among Brazilian women [15].

Despite the fact that the literature is more conclusive regarding the loss of respiratory muscle strength after 65 years of age, Chen et al [39] showed that this decrease in strength is gradual and occurs in relatively young groups of healthy subjects, especially in women [39]. These authors found that the aging effect on respiratory muscle strength started even at 30 or 40 years of age. This decrease in respiratory muscle function is probably due to a disproportionate reduction in the number of fatigue-resistant muscle fibers during the aging process [39].

There is evidence that as of the age of 65, inspiratory muscle strength declines by 0.8 to 2.7 cmH20 per year, possibly due to muscular atrophy and the loss of fast muscle fibres in the diaphragm. This loss is higher in women than in men of the same age [33, 40]. Some reasons have been suggested as possible causes, such as reduced oestrogen levels after menopause [11] and multiparity [12]. The hormonal alterations caused by the event of menopause generate a decrease in global muscle strength and an increased compression of the thoracic spine due to osteoporosis, directly interfering with the maximal inspiratory pressures [9–12]. Those are important particularities when evaluating the female aging process and justify the need to follow for the healthy women population who are going through the ageing process.

Despite the evidence regarding the high prevalence of respiratory muscle weakness and its close relationship with mobility [33], balance and even mortality [19, 32, 41], it is surprising to find that the importance of evaluating respiratory function in the clinical context is still not widely recognised. This seems to be a far from common practice in geriatric medicine. This study, like earlier ones, reinforces the need for systematic monitoring of respiratory muscle strength and its components, such as resistance, in populations considered at risk of presenting respiratory muscle weakness. Plus, it reinforces the need to assess the female population, their ageing process and particularities considering their reproductive female variables, as menopause, number of pregnancies and age of menarche, since are variable that influences directly the women global health [10–13, 15, 22].

### Implications for clinical practice

The results of this study highlight important aspects that may be useful for clinical practice, specifically for the female population. Firstly, we have identified weakness of inspiratory muscle strength, as well as poor balance at quite early ages in women, before 65 years of age. In addition, these dysfunctions were independent of the level of physical activity they perform, supporting the idea that global physical exercise does not affect the respiratory muscles. Therefore, therapeutic exercise programs widely known as "core training" must take into consideration the work of the diaphragm and other respiratory muscles, as a fundamental component of it. The scientific literature supporting inspiratory muscle training is overwhelming, reporting numerous beneficial effects, including improved static and dynamic balance [42, 43]. In addition, this training can be self-managed, has low costs and risks.

### Strengths and limitations

The current study is on a topic of relevance and shows some strengths. Once women face many health-related issues in their lifetime, due mainly to hormonal changes, this research contributes to several women’s health topics. Additionally, the study protocol used different and objective techniques to evaluate general and respiratory health issues of a specific women population, regarding the importance of the theme related to respiratory muscle strength and lung function as well as its relationship with some other systems.

This study presents some other limitations. First, due to the cross-sectional nature of this research, the establishment of causality could not be determined and longitudinal research on this subject is essential to better understand the detected relationship between balance and respiratory strength. Second, the study participants were selected by convenience sampling; the evaluation could therefore be influenced by the motivation of the participant, and thus may not be representative of the general population. Third, the electronic manometer was not calibrated since it had been bought, i.e., a year before the beginning of the study data collection.

Other confounding variables, as well as complementary sniff nasal inspiratory pressure (SNIP) measurements, could have been used to obtain more solid results on the role of the inspiratory muscles in balance, since it has been reported that the use of maximal respiratory pressure manoeuvres may be limited because of the difficulty of fitting in the mouthpiece for evaluation, especially when considering older people, and that the SNIP test could be useful to obtain additional information on inspiratory pressures [28].

## Conclusion

In conclusion, this study shows that inspiratory muscle weakness is associated with deficient postural balance in middle-aged and elderly women, even after adjustment for confounding factors. The novelty of this work is having evaluated an exclusively healthy female sample, assessed through objective tools and including middle-aged and older women. Our findings call attention to the need for multidimensional assessment and rehabilitation strategies for subjects identified as having MIP weakness and/or balance disorders. Assessing MIP among middle-aged and older women with balance disorders may help health professionals to direct appropriate rehabilitation strategies aiming at preventing disability. In the same way, assessing balance among those identified with MIP weakness may identify those in need for balance training as part of their rehabilitation. Moreover, other studies on this subject would be important, including the SNIP test when evaluating respiratory muscles strength, and considering a longitudinal approach in a new exclusively female population to better understand the relationships found in this sample.

## Take-home points

- We have identified weakness of inspiratory muscle strength, as well as poor postural balance at quite early ages in women (before 65 years of age);
- Inspiratory muscle weakness is associated with deficient postural balance in this sample;
- Inspiratory muscle weakness and deficient postural balance were independent of the physical activity level, supporting the idea that global physical exercise does not affect the respiratory muscles;
- There is a need for multidimensional assessment and rehabilitation strategies for subjects identified as having MIP weakness and/or balance disorders.

## Supporting information

S1 Questionnaire Inclusivity in global research. (DOCX)
